# The Interleukin-6 inflammation pathway from cholesterol to aging – Role of statins, bisphosphonates and plant polyphenols in aging and age-related diseases

**DOI:** 10.1186/1742-4933-4-1

**Published:** 2007-03-20

**Authors:** Sota Omoigui

**Affiliations:** 1 Division of Inflammation and Pain Medicine, L.A Pain Clinic, 4019 W. Rosecrans Ave, Los Angeles, CA 90250, USA

## Abstract

We describe the inflammation pathway from Cholesterol to Aging. Interleukin 6 mediated inflammation is implicated in age-related disorders including Atherosclerosis, Peripheral Vascular Disease, Coronary Artery Disease, Osteoporosis, Type 2 Diabetes, Dementia and Alzheimer's disease and some forms of Arthritis and Cancer. Statins and Bisphosphonates inhibit Interleukin 6 mediated inflammation indirectly through regulation of endogenous cholesterol synthesis and isoprenoid depletion. Polyphenolic compounds found in plants, fruits and vegetables inhibit Interleukin 6 mediated inflammation by direct inhibition of the signal transduction pathway. Therapeutic targets for the control of all the above diseases should include inhibition of Interleukin-6 mediated inflammation.

## Background

In 400 B.C., Hippocrates recognized the relationship between health and food. He said: "Let food be your medicine and medicine be your food". In 1513, Spanish explorer Juan Ponce de Leon discovered Florida while searching for the Fountain of Youth, a mythical spring said to restore youth. Ponce de Leon died trying to find those waters. He should have been looking instead for the Flora of Youth and inhibitors of Interleukin 6 mediated inflammation.

Aging is associated with increased frequency of several disorders including Atherosclerosis, Peripheral Vascular Disease, Coronary Artery Disease, Osteoporosis, Type 2 Diabetes, Dementia and Alzheimer's disease and some forms of Arthritis and Cancer. Aging is also characterized by a proinflammatory state that contributes to the onset of disability and age-related diseases. Proinflammatory cytokines play a central role in mediating cellular and physiological responses. Studies of the effects of aging on inflammatory response show interleukin-6 (IL-6), tumor necrosis factor-alpha (TNF-alpha) and interleukin-1beta (IL-1beta) to be important [1]. This review will focus on inhibition of Interleukin 6 mediated inflammation as key to the prevention and treatment of aging and age-related disorders.

## Atherosclerosis

Cardiovascular disease (CVD) is the leading cause of death and disability in developed nations and is increasing rapidly in the developing world. By the year 2020, it is estimated that CVD will surpass infectious diseases as the world's leading cause of death and disability. Atherosclerotic vascular disease (ASVD), which encompasses coronary heart disease, cerebrovascular disease, and peripheral arterial disease, is responsible for the majority of cases of CVD in both developing and developed countries [2]. Atherosclerosis, a progressive disease characterized by the accumulation of lipids and fibrous elements in the arteries, constitutes the single most important contributor to this growing burden of cardiovascular disease. The link between lipid metabolism and atherosclerosis dominated the thinking until the 1980s [3]. Over the last fifteen years, however, a prominent role for inflammation in the pathogenesis of atherosclerosis has been established [4]. Now atherosclerosis is considered as an inflammation-mediated disease driven by complex interactions between leukocytes, platelets and cells of the vessel wall.

Endothelial injury is the first and crucial step in the pathogenesis of atherosclerosis. A plethora of genetically determined and epigenetic factors, such as oxidized low-density lipoprotein (LDL), free radicals (e.g., due to cigarette smoking), hypertension, diabetes mellitus, elevated plasma homocysteine, infectious microorganisms, autoimmune reactions, and combinations thereof, have been identified as etiological principles. Endothelial injury triggers inflammation with increased adhesiveness and activation of leukocytes (mainly monocytes) and platelets, which is accompanied by the production of cytokines, chemokines, vasoactive molecules and growth factors.

The hallmark of the early atherosclerotic lesion is the Cholesterol ester-laden (CE-laden) macrophage foam cell [5]. Progressive "free" cholesterol (FC) loading of lesional macrophages leads to a series of phospholipid-related adaptive responses. These adaptive responses eventually fail, leading to macrophage death. Macrophage death by necrosis leads to lesional necrosis, release of cellular proteases, inflammatory cytokines, and prothrombotic molecules, which could contribute to plaque instability, plaque rupture, and acute thrombotic vascular occlusion [6]. Indeed, necrotic areas of advanced atherosclerotic lesions are known to be associated with death of macrophages, and ruptured plaques from human lesions have been shown to be enriched in apoptotic macrophages. The presence of apoptotic and necrotic macrophages in atherosclerotic lesions has been well documented in many human and animal studies [7,8].

Currently, the inflammatory mediators implicated in the pathogenesis of atherosclerosis include cytokines, chemokines, vasoactive molecules and growth factors. The anti-inflammatory effects of statins are attributed to multifaceted mechanisms including inhibition of cell cycle progression, induction of apoptosis, reduction of cyclooxygenase-2 activity and a biphasic, dose-dependent effect on angiogenesis [9]. At the center of these mechanisms stands the ability to inhibit G protein prenylation through a reduction of farnesylation and geranylgeranylation [10].

In order to advance the current theories and thinking [11], and clarify the relationship between these common illnesses, we submit our theory of the precise biochemical pathway, between cholesterol and inflammation, and between inflammation and aging and age-related disorders including Atherosclerosis, Peripheral Vascular Disease, Coronary Artery Disease, Osteoporosis, Type 2 Diabetes, Dementia and Alzheimer's disease and some forms of Arthritis and Cancer. By elaborating this biochemical pathway, we will delineate a mechanism of the pleiotropic effects of statins, bisphosphonate drugs and polyphenolic compounds. The common mechanism of action and common pleiotropic effects of the statins, bisphosphonate drugs and plant derived and synthetic polyphenolic compounds in addition to our identification of the unique activity of the Interleukin 6 cytokine among all the vast mediators of inflammation and the inflammatory response enabled us to reverse engineer this biochemical pathway. Each component of our theory is supported and validated by numerous research studies.

## Acute Phase Response

The acute phase response occurs prior to antibody-mediated immunological defense. It occurs in response to an inflammatory response brought on by injury and trauma, neoplasm, or disordered immunological activity. A local reaction at the site of injury or infection leads to an activation of cytokines (specifically, IL-6, IL-1, TNF-Alpha, and interferons) that triggers a systemic response consisting of leukocytosis; increases in glucocorticoid production; increases in erythrocyte sedimentation rates, fever, activation of complement and clotting cascades; decreases in serum zinc and iron; and an increase in plasma levels of acute phase proteins, C-reactive protein (CRP), serum amyloid A, fibrinogen, and other proteins [12].

Levels of cytokines involved in the acute phase response – TNF-Alpha, IL-1, IL-6, and fibrinogen – have been shown to be elevated in cases of unstable angina related to aneurysm [13-15] and have been positively correlated with the risk of primary and recurrent myocardial infarction and death [16-18]. The risk associated with these elevated levels remains constant even when the data is adjusted for other major risk factors: blood pressure, total and HDL cholesterol, body mass index, diabetes, alcohol use, family history, and exercise frequency [15]. Elevated levels of highly sensitive C-reactive protein (hs-CRP) have been related to increased risk of cardiovascular disease, myocardial infarction, and coronary artery disease (CAD) deaths among individuals with angina pectoris [19-21]. Assayed levels of hs-CRP can increase 100 times over normal levels within 24–48 hours after an acute inflammatory stimulus. However, in long term prospective studies inter-individual variations in hs-CRP levels may occur over long periods of time, in the absence of trauma or acute infection [22] Elevated levels of hs-CRP have shown a doubling of risk both for ischemic stroke in hypertensive men and women [14,23] and for peripheral artery disease [24].

Recent studies are now demonstrating that IL-6 and TNF-alpha are stronger predictors of cardiovascular disease than C-reactive protein. In the Health, Aging and Body Composition study [25], done at the Wake Forest University School of Medicine, the researchers tracked the medical history of the 2,225 participants for an average of 42 months after measuring their blood levels of C-reactive protein, IL-6 and TNF-alpha. People with the highest IL-6 levels were two to five times more likely to have a heart attack, stroke or other cardiovascular episode than those with the lowest levels. High blood levels of TNF-alpha increased the risk of heart disease by 79 percent and of heart failure by 121 percent. High levels of C-reactive protein increased the risk of heart failure by 160 percent compared to those with low levels, but they did not significantly raise the risk of a first stroke or heart attack.

As expected, the incidence of cardiovascular disease was high for people with the conventional risk factors – smoking, high blood pressure, high cholesterol and the like. But for participants free of those risk factors, the inflammation-related molecules were better predictors of heart disease.

## Cholesterol Metabolism

**Figure 1:**
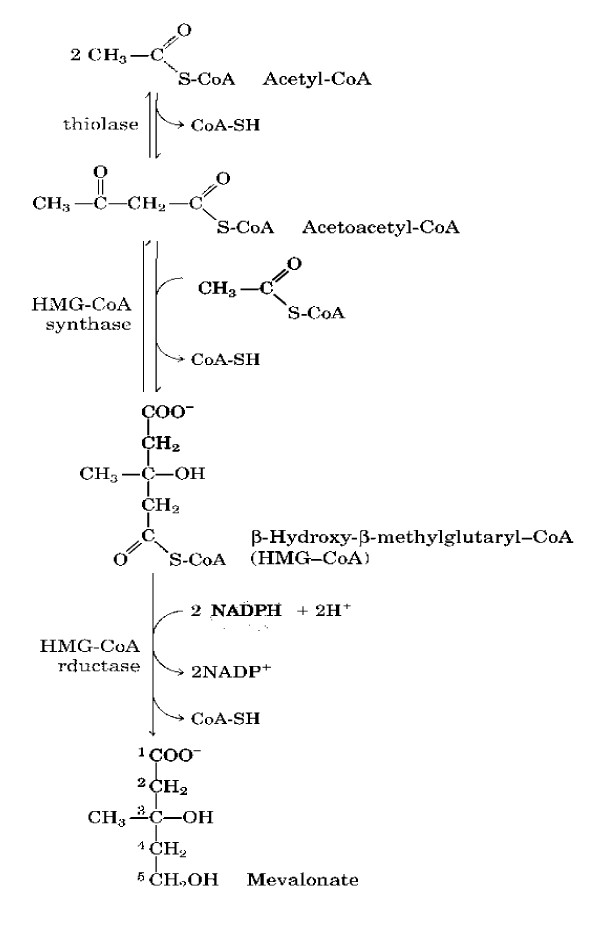
Mevalonate Synthesis.

Normal healthy adults synthesize cholesterol at a rate of approximately 1 g/day and consume approximately 0.3 g/day. A relatively constant level of cholesterol in the body (150 – 200 mg/dL) is maintained primarily by controlling the level of *de novo *synthesis. The level of cholesterol synthesis is regulated in part by the dietary intake of cholesterol. Cholesterol from both diet and synthesis is utilized in the formation of membranes and in the synthesis of the steroid hormones and bile acids. The greatest proportion of cholesterol is used in bile acid synthesis [26]. Cholesterol synthesis occurs in the cytoplasm and microsomes with initial synthesis of mevalonate from the two-carbon acetate group of acetyl-CoA. See Figure 1 (Mevalonate Synthesis).

1. Synthesis begins when acetyl-CoA is derived from an oxidation reaction in the mitochondria and is transported to the cytoplasm

2. Two moles of acetyl-CoA are condensed, forming acetoacetyl-CoA. Acetoacetyl-CoA and a third mole of acetyl-CoA are converted to 3-hydroxy-3-methylglutaryl-CoA (HMG-CoA) by the action of HMG-CoA synthase.

3. HMG-CoA is converted to mevalonate, in a rate limiting step catalyzed by the enzyme HMG-CoA reductase, (HMGR)

**Figure 2:**
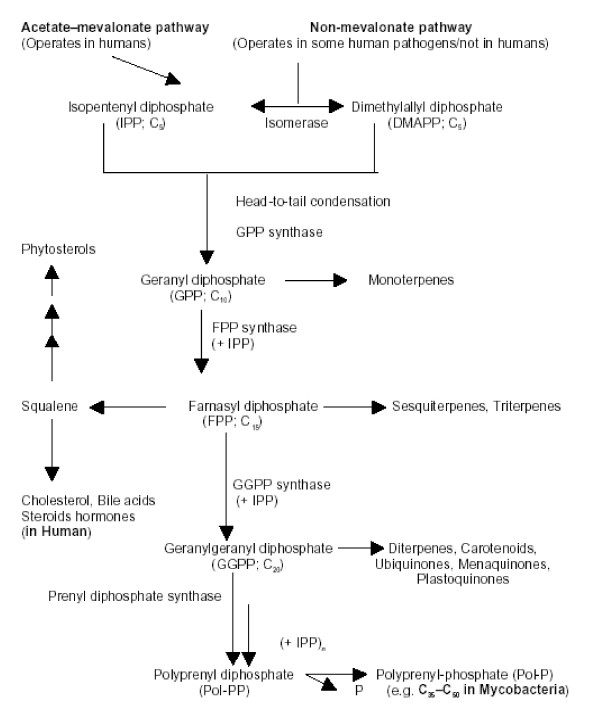
Cholesterol and Isoprenoid Synthesis [181].

In human beings, cholesterol and isoprenoids are then synthesized via the mevalonate pathway. See Figure 2 (Cholesterol and Isoprenoid Synthesis).

1. Mevalonate is activated by three successive phosphorylations, yielding 5-pyrophosphomevalonate

2. After phosphorylation, an ATP-dependent decarboxylation yields isopentenyl pyrophosphate, (IPP), an activated isoprenoid molecule. Isopentenyl pyrophosphate is in equilibrium with its isomer, dimethylallyl pyrophosphate, DMAPP.

3. One molecule of IPP condenses with one molecule of DMAPP to generate geranyl pyrophosphate, (GPP). This step is catalyzed by GPP synthase.

4. GPP further condenses with another IPP molecule to yield farnesyl pyrophosphate, (FPP). This step is catalyzed by FPP synthase.

5. FPP condenses with another IPP molecule to yield geranylgeranyl pyrophosphate (GGPP). This step is catalyzed by GGPP synthase

6. The head-to-tail condensation of two molecules of FPP yielding Squalene, is catalyzed by squalene synthase.

7. Squalene undergoes a two-step cyclization to yield lanosterol.

8. Lanosterol is converted to cholesterol, through a series of 19 additional reactions

There is a complex regulatory system to co-ordinate the biosynthesis of cholesterol with the availability of dietary cholesterol. The cellular supply of cholesterol is maintained at a steady level by the following mechanisms:

1. Regulation of HMGR activity and levels

2. Regulation of excess intracellular free cholesterol through the activity of acyl-CoA:cholesterol acyltransferase, (ACAT)

3. Regulation of plasma cholesterol levels via LDL receptor-mediated uptake and HDL-mediated reverse transport.

## Interleukin 6

The Interleukin-6 family of cytokines, signaling through the common receptor subunit (glycoprotein) subsequently activates signal transducers and activators of transcription (STAT3), mitogen-activated proteinkinase (MAPK), and phosphatidylinositol 3-kinase (PI3K) [27]. The interleukin-6 (IL6) family comprises interleukin (IL)-6, IL-11, leukemia inhibitory factor, oncostatin M, ciliary neurotrophic factor and cardiotrophin-1. Among its many functions, IL-6 plays an active role in inflammation, immunology, bone metabolism, reproduction, arthritis, neoplasia, and aging. IL-6 expression is regulated by a variety of factors, including steroidal hormones, at both the transcriptional and post-transcriptional levels. Elevated levels of IL-6 are associated with the highest risks for subclinical cardiovascular disease as well as for clinical cardiovascular disease in older men and women [28]. Elevated levels of IL-6 are associated with a 34 percent increased likelihood of cognitive decline in older men and women [29]. Interleukin-6 mediated inflammation contributes to bone resorption and osteoporosis by stimulating osteoclastogenesis and osteoclast activity [30-32]. Interleukin (IL)-6 production is considerably enhanced and associated with bone destruction in Staphylococcus aureus and mycobacterial arthritis, osteitis or osteomyelitis [33-35]. During times of stress or depression, IL-6 levels are increased. In a study of older adults undergoing a chronic stressor (men and women who were caregiving for a spouse with dementia), Caregivers' average rate of increase in IL-6 was about four times as large as that of non-caregivers [36,37].

IL-6 transmits its biological signal through two proteins on the cell. One of them is IL-6 receptor (IL-6R), an IL-6-specific binding molecule with a molecular weight of about 80 kD. The other is a membrane-bound protein gp130 having a molecular weight of about 130 kD that is involved in non-ligand-binding signal transduction. IL-6 receptor exists not only in the membrane-bound form with transmembrane domain expressed on the cell surface but also as a soluble IL-6 receptor consisting mainly of the extracellular region. IL-6 and IL-6 receptor form the IL-6/IL-6 receptor complex, which after binding to gp130 transmits its biological signal to the cell. The important participants in the Interleukin-6 signaling pathway include the Janus kinases (JAKs) Jak1, Jak2 and Tyk2, the signal transducers and activators of transcription STAT1 and STAT3, the tyrosine phosphatase SHP2 [SH2 (Src homology 2) domain-containing tyrosine phosphatase] and transcription factor NF-κB.

## Protein Kinases

Engagementof cell surface Interleukin-6 receptors activates the Janus kinase(JAK) family of tyrosine kinases, which in turn phosphorylate the cytoplasmic part of gp130, thereby creating docking sites for STAT factors STAT1 and STAT3 [38,39]. Activated STATs dimerize upon activation by JAKs and translocate to the nucleus where theybind specific DNA response elements and regulate the expressionof certain genes. Following gp130 dimerization, IL-6 activates multiple signaling pathways (Ras dependent MAP Kinase cascade, STAT1-STAT3 heterodimer pathway, and STAT3 homodimer pathway) [40-42].

## Dimeric transcription factors

Activator protein-1 (AP-1) is a collective term referring to dimeric transcription factors composed of Jun, Fos, or ATF (activating transcription factor) subunits that bind to the AP-1 binding site on the several proinflammatory genes including the IL-6 promoter [43]. AP-1 activity plays an important role in the inflammatory response by modulating gene expression of several inflammatory mediators including IL-6 transcription. Phosphorylation of c-Jun is a prerequisite of AP-1 dimerization and activation. AP-1 activity is controlled by signaling through the JNK family of MAP kinases. It has been demonstrated that during reperfusion, oxidative stress leads to activation and translocation of JNK to the nucleus, where phosphorylation of transcription factors, such as c-Jun occurs.

## Nuclear factor kappa b

Nuclear factor κB (NF-κB) is a widely expressed, inducible transcription factor of particular importance to cells of the immune system. It was originally identified as an enhancer binding protein for the Ig κ-light chain gene in B cells [44]. NF-κB regulates the expression of many genes involved in mammalian immune and inflammatory responses, including cytokines, cell adhesion molecules, complement factors, and a variety of immunoreceptors. The NF-κB transcription factor is a heterodimeric protein that comprises the p50 and p65 (Rel A) subunits. These subunits are proteins of the Rel family of transcriptional activators. Members of the Rel family share a conserved 300-amino acid Rel homology domain responsible for DNA binding, dimerization, and nuclear localization. While transcriptionally active homodimers of both p50 and p65 can form, the p50/65 heterodimer is preferentially formed in most cell types [45].

In the absence of stimulatory signals, the NF-κB heterodimer is retained in the cytoplasm by its physical association with an inhibitory phosphoprotein, IκB. Multiple forms of IκB have been identified [46]. Two of these forms, IκBα and IκBβ, have been shown to modulate the function of the NF-κB heterodimer, and these two IκBs are phosphorylated in response to different extracellular stimuli [47]. Recent studies indicate that the catalytic subunit of protein kinase A (PKA_C_) is associated with the NF-κB/IκBα complex [48]. In this p50/p65/IκBα/PKA_C _tetrameric configuration, IκBα renders PKA_C _inactive and masks the nuclear localization signal on NF-κB. Proinflammatory stimuli can activate a number of protein kinases, which have the capacity to modulate nuclear factor-κB (NF-κB) or activator protein-1 (AP-1) activity. A variety of extracellular stimulatory signals, such as cytokines, viruses, and oxidative stressors [49] activate kinases that phosphorylate IκB. The cytokine-activated IκB kinase termed IKK is the key regulatory kinase for IκBα [50]. IkappaB kinase (IKK) complex is composed of subunits, IKK-alpha, IKK-beta and IKK-gamma, which are serine/threonine protein kinases whose function is needed for NF-kappaB activation by pro-inflammatory stimuli [51]. Phosphorylation at serines 32 and 36 targets IκBα for ubiquitination and subsequent rapid proteolysis via a proteasome-mediated pathway [52-55], resulting in the release of NF-κB/PKA_C_. The now active PKA_C _subunit dissociates and phosphorylates the p65 subunit of NF-κB. Phosphorylated NF-κB then translocates to the cell nucleus, where it binds to target sequences in the chromatin and activates specific gene subsets, particularly those important to immune and inflammatory function [56-58]. PPAR alpha (Peroxisome proliferator-activated receptor alpha) negatively interferes with inflammatory gene expression by up-regulation of the cytoplasmic inhibitor molecule IkappaB alpha, thus establishing an autoregulatory loop. This induction takes place in the absence of peroxisome proliferator-response elements (PPRE), but requires the presence of NF-kappaB and Sp1 elements in the IkappaB alpha promoter sequence as well as DRIP250 cofactors [59].

Nuclear factor-kappaB (NF-kappaB) is a required transcription factor for Ang II-inducible IL-6 expression. Interleukin-6 (IL-6) is expressed by angiotensin II (Ang II)-stimulated vascular smooth muscle cells (VSMCs). In one study Ang II treatment induced IL-6 transcription by inducing cytoplasmic-to-nuclear translocation of the NF-kappaB subunits Rel A and NF-kappaB1 with parallel changes in DNA-binding activity in a biphasic manner, which produced an early peak at 15 minutes followed by a nadir 1 to 6 hours later and a later peak at 24 hours [60].

## Peroxisome Proliferator-Activated Receptors (PPARs)

Peroxisome proliferator-activated receptors (PPARs) are ligand-activated transcription factors which form a subfamily of the nuclear receptor gene family. The PPAR subfamily consists of three isotypes, alpha (NR1C1), gamma (NR1C3), and beta/delta (NRC1C2) with a differential tissue distribution. PPARs are activated by ligands, such as naturally occurring fatty acids, which are activators of all three PPAR isotypes. In addition to fatty acids, several synthetic compounds, such as fibrates and thiazolidinediones, bind and activate PPARalpha and PPARgamma, respectively. PPARalpha is expressed primarily in tissues with a high level of fatty acid catabolism such as liver, brown fat, kidney, heart and skeletal muscle. PPARbeta is ubiquitously expressed, and PPARgamma has a restricted pattern of expression, mainly in white and brown adipose tissues, whereas other tissues such as skeletal muscle and heart contain limited amounts. Furthermore, PPARalpha and gamma isotypes are expressed in vascular cells including endothelial and smooth muscle cells and macrophages/foam cells. In order to be transcriptionally active, PPARs need to heterodimerize with the retinoid-X-receptor (RXR). Upon activation, PPAR-RXR heterodimers bind to DNA specific sequences called peroxisome proliferator-response elements (PPRE) and stimulate transcription of target genes. PPARs play a critical role in lipid and glucose homeostasis, but lately they have been implicated as regulators of inflammatory responses. The first evidence of the involvement of PPARs in the control of inflammation came from the PPARalpha null mice, which showed a prolonged inflammatory response. PPARalpha activation results in the repression of NF-kappaB signaling and inflammatory cytokine production in different cell-types. A role for PPARgamma in inflammation has also been reported in monocyte/macrophages, where ligands of this receptor inhibited the activation of macrophages and the production of inflammatory cytokines (TNFalpha, interleukin 6 and 1beta) [61]. PPAR activators have effects on both metabolic risk factors and on vascular inflammation related to atherosclerosis. PPAR have profound effects on the metabolism of lipoproteins and fatty acids. PPAR alpha binds hypolipidemic fibrates, whereas PPAR gamma has a high affinity for antidiabetic glitazones. Both PPAR alpha and gamma are activated by fatty acids and their derivatives. Activation of PPAR alpha increases the catabolism of fatty acids at several levels. In the liver, it increases uptake of fatty acids and activates their beta-oxidation. The effects that PPAR alpha exerts on triglyceride-rich lipoproteins is due to their stimulation of lipoprotein lipase and repression of apolipoprotein CIII expression, while the effects on high-density lipoproteins depend upon the regulation of apolipoproteins AI and AII. PPAR gamma has profound effects on the differentiation and function of adipose tissue, where it is highly expressed. PPAR are also expressed in atherosclerotic lesions and are present in vascular endothelial cells, smooth muscle cells, monocytes, and monocyte-derived macrophages. Via negative regulation of nuclear factor-kappa B and activator protein-1 signaling pathways, PPAR alpha inhibits expression of inflammatory genes, such as interleukin-6, cyclooxygenase-2, and endothelin-1. Furthermore, PPAR alpha inhibits expression of monocyte-recruiting proteins such as vascular cell adhesion molecule (VCAM)-1 and induces apoptosis in monocyte-derived macrophages. PPAR gamma activation in macrophages and foam cells inhibits the expression of activated genes such as inducible nitric oxide synthase, matrix metalloproteinase-9 and scavenger receptor A. PPAR gamma may also affect the recruitment of monocytes in atherosclerotic lesions as it is involved in the expression of VCAM-1 and intracellular adhesion molecule-1 in vascular endothelial cells[62].

## Activation of Interleukin-6 inflammation by isoprenoids

Cytokine receptors act through a complex signaling network involving GTPase proteins such as Ras, Rho, Rac, and Rab (particularly Rho), Janus kinases (JAKs) and the signal transducers and activators of transcription (STATs) to regulate diverse biological processes controlling immune function, growth, development and homeostasis [63].

Isoprenoids are necessary for posttranslational lipid modification (prenylation) and, hence, the function of Ras and other small guanosine triphosphatases (GTPases) [64].

GTPase proteins such as Ras, Rho, Rac, and Rab (particularly Rho) are intracellular signaling proteins that, when activated, are involved in receptor-coupled transduction of signals from extracellular stimuli to cytoplasm and the nucleus. Small GTPase proteins constitute a Ras superfamily, which is comprised of at least five major branches. Members of the Ras branch include the Ras, Rap, Ral and R-Ras family proteins [65,66]. The Ras family regulates gene expression. The Rho branch constitutes a second major branch, with RhoA, Rac1 and Cdc42 the most studied members. The Rho family regulates cytoskeletal reorganization and gene expression. The Rab branch is the largest, and, together with members of the Arf/Sar branch, serve as regulators of intracellular vesicular transport. Ran is the sole member of its branch and is a crucial regulator of nucleo-cytoplasmic transport of proteins and RNA. The Ras superfamily proteins alternate between an inactivated GDP-bound form and activated GTP-boundform, allowing them to act as molecular switches for growth and differentiation signals. Prenylation is a process involving the binding of hydrophobic isoprenoid groups consisting of farnesyl or geranylgeranyl residues to the C-terminal region of Ras protein superfamily. Farnesyl pyrophosphate (FPP) and Geranylgeranyl pyrophosphate (GPP) are metabolic products of mevalonate that are able to supply prenyl groups. The prenylation is conducted by prenyl transferases. The hydrophobic prenyl groups are necessary to anchor the Ras superfamily proteins to intracellular membranes so that they can be translocated to the plasma membrane [67]. The final cell-membrane fixation is necessary for Ras proteins to participate in their specific interactions [68,69]. The activity of the small GTPase, Rac1, plays a role in various cellular processes including cytoskeletal rearrangement, gene transcription, and malignant transformation. Small GTPases of the Ras protein superfamily stimulate the tyrosine phosphorylation and activation of the JAK family of intracellular kinases. This in turn activates the STAT family of transcription factors and results in the induction of Interleukin-6 and IL-6 receptor gene. Persistent Rac1 activity leads to the autocrine production and signal transduction of Interleukin-6 [36]. IL-6 itself may produce a delayed phosphorylation and activation of STAT3, and the JAK/STAT3 pathway is an indirect target of Ras and Rho GTPases [70]. Blocking the IL-6 signaling pathway inhibits Rac1-mediated STAT3-dependent gene expression. In one study [71], constitutively active Rac1 (Rac V12) is shown to stimulate the activation of STAT3. The activity of Rac1 leads to STAT3 translocation to the nucleus coincident with STAT3-dependent gene expression [72]. Rac1 expression results in the induction of the IL-6 and IL-6 receptor genes and neutralizing antibodies directed against the IL-6 receptor block Rac1-induced STAT3 activation. Inhibition of nuclear factor-kappaB activation or disruption of IL-6-mediated signaling through the expression of IkappaBalpha S32AS36A and suppressor of cytokine signaling 3, respectively, blocks Rac1-induced STAT3 activation. The study also investigated whether the other Rho family members mediate STAT3 activation in an IL-6-dependent pathway. The expression of constitutively active RhoG, Cdc42, and RhoA caused the translocation from the cytoplasm to the nucleus of cotransfected STAT3-GFP. This GTPase-induced STAT3 translocation was blocked to varying degrees by neutralizing IL-6 receptor antibodies, supporting a role for autocrine IL-6 in Rho family-induced STAT3 activation. These findings elucidate a mechanism dependent on the induction of an autocrine IL-6 activation loop through which Rac1 and the Rho family mediate STAT3 activation establishing a link between GTPase activity and Janus kinase/STAT signaling. Interestingly, STAT3 is persistently activated in many human cancers and transformed cell lines. In cell culture, active STAT3 is either required for transformation, enhances transformation, or blocks apoptosis.

In one study [73], leukemic cells from 50 patients with acute myeloid leukemia (AML) were analyzed for the presence of activating point mutations of the N-RAS gene using polymerase chain reaction (PCR) and differential oligonucleotide hybridization. Clonal activation of N-RAS, noted in the large majority of leukemic cells of the six of these patients, was correlated significantly (p = 0.0003) with the ability of these cells to express interleukin 6 (IL-6), previously shown to be expressed at high levels in approximately 30% of primary AML cells.

In summary, isoprenoids farnesyl pyrophosphate (FPP) and geranylgeranyl pyrophosphate (GPP) are necessary for posttranslational lipid modification (prenylation) and, hence, the function of Ras and other small GTPase proteins such as Ras, Rho, Rac, and Rab [52]. Persistently active Rho family and Rac1 results in the activation of JAKs and subsequent tyrosine phosphorylation and activation of STAT3 [74]. Tyrosine phosphorylated STAT3 forms dimers that translocate to the nucleus to bind DNA target sites in responsive genes [59]. IL-6 and IL-6 receptor gene induction occurs as a result of activated STAT proteins and IL-6 mediates the long-term activation of STAT3 through an autocrine loop.

## Inhibition of cholesterol pathway by statins

The main effect of statins is the decrease of serum level of low-density lipoprotein (LDL) cholesterol, due to the inhibition of intracellular cholesterol biosynthesis. A minor effect is the decrease of serum triglycerides. Statins inhibit HMG-CoA reductase and decrease the production of mevalonate, geranyl pyrophosphate, and farnesyl pyrophosphate, and subsequent products on the way to construction of the cholesterol molecule. Thus, statins could inhibit inflammation, by inhibition of the cholesterol pathway and intracellularly interfering with Ras superfamily protein function [75]. Ikeda *et al*. [76] recently showed that statins decrease matrix metalloproteinase-1 expression through inhibition of Rho. Statin therapy has been demonstrated to provide significant reductions in non-high-density lipoprotein cholesterol, and to decrease cardiovascular morbidity and mortality.

## Inhibition of cholesterol pathway by bisphosphonates

Recent findings suggest that alendronate and other N-containing bisphosphonates inhibit the isoprenoid biosynthesis pathway and interfere with protein prenylation, as a result of reduced geranylgeranyl diphosphate levels. One study [77] utilizing High-performance liquid chromatography (HPLC) analysis of products from a liver cytosolic extract, identified farnesyl disphosphate (FPP) synthase as the mevalonate pathway enzyme inhibited by bisphosphonates. Recombinant human farnesyl diphosphate synthase was inhibited by alendronate with an IC(50) of 460 nM (following 15 min preincubation). Alendronate did not inhibit isopentenyl diphosphate isomerase or GGPP synthase. Recombinant farnesyl diphosphate synthase was also inhibited by pamidronate (IC(50) = 500 nM) and risedronate (IC(50) = 3.9 nM), negligibly by etidronate (IC50 = 80 microM), and not at all by the non-nitrogen-containing bisphosphonate clodronate. In another study, a wide range of bisphosphonates, were found to have a significant correlation between potency for inhibition of recombinant human FPP synthase *in vitro *and anti-resorptive potency *in vivo*, suggesting that this enzyme is the major pharmacologic target of these drugs. The most potent anti-resorptive bisphosphonates such as zoledronic acid and risedronate are very potent inhibitors of FPP synthase, with IC50 values as low as 3 nM and 10 nM respectively. Inhibition of FPP synthase prevents the formation of FPP and its derivative GGPP. These isoprenoid lipids are necessary for the post-translational lipid modification (prenylation) of small GTPase proteins such as Ras, Rho, Rac, and Rab. The effects of nitrogen-containing bisphosphonates on osteoclasts can be overcome by addition of components of the mevalonate pathway, which bypass the inhibition of FPP synthase and restore protein prenylation. In particular, geranylgeraniol (a cell-permeable form of GGPP) prevents inhibition of resorption by nitrogen-containing bisphosphonates *in vitro *[78].

## Fungi, plant-derived polyphenolic compounds and fatty acids

Statins identical to the cholesterol lowering pharmaceutical lovastatin and its derivatives of simvastatin, pravastatin and mevastatin can be produced by a variety of filamentous fungi, including Monascus, Aspergillus, Penicillium, Pleurotus, Pythium, Hypomyces, Paelicilomyces, Eupenicillium, and Doratomyces [79]. As a food product, rice fermented with a red Monascus fungus (red rice) has been known to contain low amounts of statins and used for hundreds of years in China. Red rice is used in wine making, as a food-coloring agent and as a drug in traditional Chinese medicine.

Several hundred molecules having a polyphenol (polyhydroxyphenol) structure (i.e. several hydroxyl groups on aromatic rings) have been identified in edible plants. These molecules are secondary metabolites of plants and are generally involved in defense against ultraviolet radiation or aggression by pathogens. Polyphenols are widespread constituents of fruits, vegetables, cereals, dry legumes, chocolate, and beverages, such as tea, coffee, or wine. These compounds may be classified into different groups as a function of the number of phenol rings that they contain and of the structural elements that bind these rings to one another. Classes of polyphenols include the phenolic acids, flavonoids, stilbenes, and lignans. There are two classes of phenolic acids: derivativesof benzoic acid and derivatives of cinnamic acid.

Hydroxybenzoic acids are components of complex structures such as hydrolyzable tannins (gallotanninsin mangoes and ellagitannins in red fruit such as strawberries, raspberries, and blackberries). Hydroxycinnamic acids are more common than are the hydroxybenzoicacids and consist chiefly of *p*-coumaric, caffeic, ferulic, and sinapic acids. Caffeic and quinic acid combine to form chlorogenic acid, whichis found in many types of fruit and in high concentrations in coffee.

Flavonoids, are the largest single class as far as total numbers of known compounds. About two-thirds of the polyphenols we obtain in our diets are flavonoids. Flavonoids share a common structure consisting of 2 aromatic rings that are bound together by 3 carbon atoms that form an oxygenated heterocycle, and may be divided into 6 major subclasses: Anthocyanidins (e.g., cyanidin, pelargonidin); Flavanols (e.g., epicatechin, gallocatechin); Flavones (e.g., apigenin, luteolin); Flavonols (e.g., kaempferol, myricetin, quercetin); Flavanones (e.g., hesperidin, naringenin); Isoflavones (e.g., genistein, daidzein, biochanin) and Proanthocyanidins [80].

Proanthocyanidins (condensed tannins) are a class of polyphenolic compounds found in several plant species. They include procyanidins, which are chains of catechin, epicatechin, and their gallic acid esters and the prodelphinidins, which consist of gallocatechin, epigallocatechin, and their gallic acid esters as the monomeric units.

Isoflavones are flavonoids with structural similarities to estrogens. Although they are not steroids, they have hydroxyl groups in positions 7 and 4 in a configuration analogous to that of the hydroxyls in the estradiol molecule. This confers pseudohormonal properties on them, including the ability to bind to estrogen receptors, and they are consequently classified as phytoestrogens. Phytoestrogenic isoflavones including genistein, daidzein, glycitein, biochanin A, formononetin, and their respective naturally occurring glycosides and glycoside conjugates are found in plants such as legumes, clover, and the root of the kudzu vine (pueraria root). Common legume sources of these isoflavone compounds include soy beans, chick peas, ground nuts, lentils and various other types of beans and peas. Clover sources of these isoflavone compounds include red clover and subterranean clover.

Fatty acids consist of chains of carbon atoms linked together by chemical bonds. Fatty acids come in different lengths: short chain fatty acids have fewer than 6 carbons, while long chain fatty acids have 12 or more carbons. On one terminal of the carbon chain is a methyl group and on the other terminal is a carboxyl group. The chemical bonds between the carbon atoms determine whether a fatty acid is saturated or unsaturated. Saturated fatty acids contain single bonds only. Examples of foods high in saturated fats include lard, butter, whole milk, cream, eggs, red meat, chocolate, and solid shortenings. An excess intake of saturated fat can raise blood cholesterol and increase the risk of developing coronary heart disease. Monounsaturated fatty acids contain one double bond. Examples of foods high in monounsaturated fat include avocados, nuts, and olive, peanut, and canola oils. Polyunsaturated fatty acids contain more than one double bond. Examples of foods high in polyunsaturated fats include vegetable oils, corn, sunflower, and soy. Essential fatty acids are polyunsaturated fatty acids that the human body needs for metabolic functioning but cannot produce, and therefore has to be acquired from food. Omega-3 fatty acids are a class of essential polyunsaturated fatty acids with the double bond in the third carbon position from the methyl terminal (hence the use of "3" in their description). Foods high in omega-3 fatty acids include cold-water fatty fish such as salmon, herring, mackerel, anchovies and sardines, and vegetable sources such as the oil from the seeds of chia, perilla, flax, purslane, hemp, and canola. Other foods that contain omega-3 fatty acids include whole grains, beans, green leafy vegetables such as spinach and seafood such as shrimp, clams, light chunk tuna, catfish and cod. Omega-6 fatty acids are a class of essential polyunsaturated fatty acids with the initial double bond in the sixth carbon position from the methyl group. Examples of foods rich in omega-6 fatty acids include corn, safflower, sunflower, soybean, and cottonseed oil. Omega-3 and omega-6 fatty acids are also referred to as n-3 and n-6 fatty acids, respectively.

## Atherosclerosis and Interleukin 6

Macrophage uptake of oxidized low-density lipoprotein (Ox-LDL) is a hallmark of the early atherosclerotic lesion, and may be mediated by Interleukin-6. Incubation of IL-6 with MPM or IL-6 administration in mice increased macrophage Ox-LDL degradation and CD36 mRNA expression. Angiotensin II (Ang II) plays an important role in atherogenesis. Ang II increases macrophage cholesterol accumulation and foam cell formation, increases contraction of blood vessels and induces hypertrophyand hyperplasia of vascular smooth muscle cells (VSMC). Ang II significantly increases the expression of IL-6 mRNA and protein in vascular smooth muscle, in a dose-dependent manner. The induction of IL-6 expression by Ang II is dependent on intracellular Ca^2+^, tyrosine phosphorylation, and mitogen-activated proteinkinase (MAPK)[81]. Ang II administration to apolipoprotein E-deficient atherosclerotic mice increases Ox-LDL degradation, CD36 mRNA expression, and CD36 protein expression by their peritoneal macrophages (MPMs). Ang II treatment of IL-6-deficient mice did not affect their MPM Ox-LDL uptake and CD36 protein levels. Furthermore, injection of IL-6 receptor antibodies in mice during Ang II treatment reduced macrophage Ox-LDL uptake and CD36 expression [82].

Enzymatic, nonoxidative modification transforms low density lipoprotein (LDL) to an atherogenic molecule (E-LDL) that activates complement and macrophages and is present in early atherosclerotic lesions. E-LDL accumulates in human vascular smooth muscle cells (VSMC), where it stimulates the expression of gp130, the signal-transducing chain of the IL-6 receptor (IL-6R) family, and the secretion of Interleukin-6 [83]. IL-6/sIL-6R provokes marked up-regulation of gp130 mRNA and surface protein expression in VSMC. This is accompanied by secretion of IL-6 by the cells, so that an autocrine stimulation loop is created. In the wake of this self-sustaining system, there is a selective induction and secretion of monocyte chemotactic protein-1 (MCP-1), up-regulation of ICAM-1, and marked vascular smooth muscle proliferation [84]. Interleukin-6 (IL-6) induces proliferation of vascular smooth muscle cells and the release of monocyte chemoattractant protein-1 (MCP-1) [85]. One study investigated IL-6 mRNA expression in atherosclerotic arteries from patients undergoing surgical vascularization, utilizing reverse transcription polymerase chain reaction (RT-PCR) and in situ hybridization analyses. In RT-PCR analysis, the atherosclerotic arteries showed 10- to 40-fold levels of IL-6 mRNA expression over the non-atherosclerotic artery. In in-situ hybridization analysis, IL-6 gene transcripts were observed in the thickened intimal layer of atherosclerotic lesions. These results strongly suggest the involvement of IL-6 in the development of human atherosclerosis [86]. Thrombin is a potent mitogen for vascular smooth muscle cells (VSMCs) and plays an important role in the progression of atherosclerosis. Thrombin induces IL-6 mRNA and protein expression in a dose-dependent manner. Pharmacological inhibition of extracellular signal-regulated protein kinase (ERK), p38 mitogen-activated protein kinase (MAPK), or epidermal growth factor receptor (EGF-R) suppresses thrombin-induced IL-6 expression [87]. IL-6 increases the number of plateletsin the circulation [88] and activates platelets through arachidonic acid metabolism in vitro [89] IL-6 is reported to increaseplasma fibrinogen and decrease free protein S concentration. These IL-6-induced modifications of platelet and the coagulant phase of the clotting mechanism may lead to pathological thrombosis and instability of plaque [90]. IL-6 stimulation of vascular smooth muscle cells occurs via the JAK/STAT signaling pathway. In one study, Rat VSMC were stimulated with IL-6 in the presence or absence of a JAK 2 inhibitor, and the activation of STAT 3 (by Western), MCP-1 (by ELISA) and DNA synthesis (by (3)H-thymidine incorporation) was determined. IL-6 rapidly induced phosphorylation of STAT 3 in a dose- and time-dependent manner with a peak expression at 30 min. IL-6 also stimulated MCP-1 protein production and DNA synthesis dose dependently. 50 microM of AG490, a specific JAK 2 inhibitor, partially inhibited STAT 3 activation and MCP-1 production, with near complete inhibition of DNA synthesis [91]. Levels of IL-6 are significantly higher in patients with dyslipidemia IIa and IIb biochemically confirmed, and IL-6 levels are significantly correlated to intima-media complex thickness [92].

## Statins and Interleukin 6

The ability of HMG-CoA reductase inhibitors to lower C-reactive protein levels has recently brought into question the mechanisms of action of the statin drugs. Because these medications lower incidences of acute cardiovascular events as well as decreasing morbidity and mortality well before the effects of lowered LDL cholesterol can be expected to occur, questions have been asked about whether they may work independently of LDL-lowering mechanisms. One study examined the effects of atorvastatin on soluble adhesion molecules, interleukin-6 (IL-6) and brachial artery endothelial-dependent flow mediated dilatation (FMD) in patients with familial (FH) and non-familial hypercholesterolemia (NFH) [93]. A total of 74 patients (27 FH and 47 NFH) were recruited. Fasting lipid profiles, soluble intercellular adhesion molecule-1 (sICAM-1), soluble vascular-cellular adhesion molecule-1 (sVCAM-1), E-selectin, IL-6 and FMD were measured at baseline, 2 weeks, 3 and 9 months post-atorvastatin treatment (FH – 80 mg/day, NFH – 10 mg/day). In both groups, compared to baseline, sICAM-1 levels were significantly reduced at 2 weeks, further reduced at 3 months and maintained at 9 months (P < 0.0001). The IL-6 levels were significantly reduced at 3 months and 9 months compared to baseline for FH (P < 0.005) and NFH (P < 0.0001). In both groups, the FMD at 2 weeks was higher than baseline (P < 0.005), with progressive improvement up to 9 months. FMD was negatively correlated with sICAM-1 and IL-6.

## Bisphosphonates and Interleukin 6

Because of various modes of action observed in studies, bisphosphonates have been classified into two groups. Bisphosphonates (such as clodronate and etidronate) that closely resemble pyrophosphate – a normal byproduct of human metabolism – are incorporated into adenosine triphosphate (ATP) analogues, which create compounds that are believed to build up and lead to osteoclast death [94]. The newest generation of bisphosphonates, which contain nitrogen (such as pamidronate, alendronate, risedronate, and ibandronate), are believed to inhibit protein prenylation (post-translational modification) within the mevalonate pathway. The mevalonate pathway is responsible for the biosynthesis of cholesterol, other sterols, and isoprenoid lipids. Isoprenoid lipids are key in the prenylation of intracellular signaling proteins (GTPases) that, when activated, regulate a number of processes, including osteoclast activity. It is believed that by impeding the function of these regulatory proteins, bisphosphonates block osteoclast functioning and cause apoptosis [95].

In patients with Paget's disease of bone, bisphosphonate therapy is associated with a significant reduction of Interleukin-6 soluble receptor (sIL-6R) serum levels [96]. Bisphosphonates inhibit the production of pro-inflammatory cytokine interleukin-6 in tumoral cell lines of human osteoblastic phenotype (MG63 and SaOs cells), and in peripheral blood mononuclear cells (PBMC) [97]. Bisphosphonates also inhibit IL-1 and TNF-alpha stimulated IL-6 release in cultures of human osteoblastic osteosarcoma cells [98]. Osteoblasts exposed to small amounts of bisphosphonate elaborate a soluble inhibitor, which interferes with osteoclast formation and development [99]. Bisphosphonates prevent apoptosis of murine osteocytic MLO-Y4 cells, whether it is induced by etoposide, TNF-alpha, or glucocorticoid dexamethasone [100]. Pamidronate and other bisphosphonates inhibit the production by osteoblasts of the inflammatory cytokine interleukin-6, a growth factor essential to myeloma cells [101].

## Plant polyphenols, fatty acids and Interleukin 6

The beneficial skeletal effects of genistein, at dietarily achievable levels, are mediated, by Interleukin-6. Interleukin-6 production was decreased 40% to 60% in osteoblastic cells treated with genistein from either day 8–16 or day 12–16, at dietarily achievable concentrations (10(-10) to 10(-8) M) (P < 0.05) [102]. In one study, Sophoricoside (SOP) an isoflavone glycosid isolated from immature fruits of Sophora japonica (Leguminosae family) inhibited the interleukin (IL)-6 bioactivity with an IC50 value of 6.1 microM [103]. In another study, treatment with soybean isoflavones (10(-5) M), in the presence of TNF-alpha (10(-10) M), for 48 h inhibited production of IL-6 and PGE(2). The authors suggested that the antiresorptive action of soy phytoestrogen may be mediated by decreases in these local factors [104]. One study investigated the mechanisms of drug resistance associated with the human prostate carcinoma PC-3 cell line. Endogenous and exogenous IL-6 and exogenous OM up-regulated cell growth and enhanced resistance of PC-3 tumor cells to both etoposide and cisplatin. Both IL-6- and OM-mediated effects were inhibited by the treatment of PC-3 with an antisense oligodeoxynucleotide against gp130, the protein kinase inhibitor genistein (GNS), or the monoterpene perillic acid (PA), a posttranslational inhibitor of p21ras isoprenylation [105]. In another study, the effect of inhibition of tyrosine kinase activity on thymidine uptake into cultured human pituitary adenoma cells was studied using two inhibitors, genistein and methyl-2,3-dihydroxycinnamate (MDHC). Of 33 pituitary adenomas, 7 incorporated sufficient [3H]thymidine to be investigated in the experiments. Genistein and MDHC both potently inhibited thymidine uptake into these tumors, with a mean inhibition by 74 mumol/L genistein of 61.96 +/- 18.96% (+/- SD inhibition of basal), by 740 mumol/L genistein of 92.65 +/- 8.59%, and by 100 mumol/L MDHC of 93.84 +/- 3.85%. Epidermal growth factor stimulated thymidine uptake in 2 of the 3 clinically nonfunctioning adenomas studied, and this stimulation was inhibited by genistein. The authors concluded that tyrosine kinase activity is crucial for the integrity and growth of pituitary adenomas in culture and that growth factors released by pituitary adenomas potentially may maintain and promote tumor growth by stimulating tyrosine kinase activity [106].

Bacterial LPS induce a 12- to 16-fold increase in IL-1 beta, IL-6, and TNF-alpha mRNA levels. In one study, this increase was completely or more than 80% blocked by the protein tyrosine kinase specific inhibitors herbimycin A and genistein at the concentrations of 1.7 and 37 microM, respectively. LPS-induced IL-6 protein synthesis and IL-6 bioactivity were also reduced to baseline levels by the PTK inhibitors herbimycin A and genistein. Both PTK inhibitors also reduced the LPS activation of nuclear factor-kappa B (NF-kappa B), which is a transcription factor involved in the expression of cytokine genes such as IL-6 and TNF-alpha [107].

Epidemiological evidence suggests that tea consumption may have a strong effect on cardiovascular disease, but there has been no prior description of the molecular mechanisms involved. Epigallocatechin-3-gallate (EGCG) is a prominent catechin present in green tea. Several experimental studies have reported beneficial effects of EGCG in inflammation and cancer [108-110]. NF-κB, is a transcription factor centrally involved in the signal transduction of the inflammatory process. The common pathway for activation of NF-κB involves phosphorylation of its inhibitor protein IκB-α by IKK. Activation of IKK complex is an essential step for NF-κB activation because the kinase phosphorylates IκB-α and allow its degradation. Several studies have demonstrated that EGCG is an effective inhibitor of IKK activity. EGCG inhibits TNF-α-mediated IKK activation in human epithelial cells. Yang and colleagues showed that EGCG in concentrations of 50 to 200 μM inhibited IKK activity in an intestinal epithelial cell line [111]. In the Myocardial ischemia reperfusion study, EGCG reduced reperfusion-induced activation of IKK, degradation of IκB-α, and activation of NF-κB [112]. EGCG has been demonstrated to dramatically inhibit chemokine induced neutrophil chemotaxis in vitro [113]. Tea polyphenols have also been noted to induce apoptosis and cell cycle arrest in a wide array of cell lines [114-116]. EGCG affects several signaling mechanisms in inflammation. Menegazzi and colleagues showed that interferon-γ-induced STAT-1 activation in carcinoma-derived cell lines of non-gut origin was blocked by EGCG [117]. In another study, Watson and colleagues demonstrated that EGCG significantly reduced INF-γ-induced STAT1 activation in T84 epithelial and THP-1 monocytes/macrophages [118].

Polyunsaturated omega-3 fatty acids reduce the secretion of proinflammatory cytokines and down regulate the inflammatory process. 18-week n-3 PUFA diet supplementation exerts a significant inhibitory effect on basal and lipopolysaccharide (LPS)-stimulated IL-6 monocyte production (50% and 46%, respectively, P < 0.05) [119,120].

## Atherosclerosis and statins

Changes in intima-media thickness (IMT) and arterial lumen diameter-as measured by B-mode high-resolution ultrasonography and quantitative coronary angiography, respectively-are currently the only surrogate markers for progression of atherosclerotic disease. There has been increasing use of this imaging technique in observational studies and interventional studies of lipid-lowering agents over the last decade. These observational studies clearly demonstrated an association between carotid IMT and atherosclerotic disease. Of the interventional studies, the recent Arterial Biology for the Investigation of the Treatment Effects of Reducing Cholesterol (ARBITER) trial found that use of atorvastatin 80 mg daily for aggressive lowering of plasma low-density lipoprotein cholesterol (LDL-C) concentrations to below current target levels was associated with significant IMT regression compared with results obtained with less aggressive plasma LDL-C lowering [121,122].

## Atherosclerosis and bisphosphonates

In one study the effect of etidronate treatment on carotidarterial intima-media thickness was prospectively examined in 57 subjects with type 2 diabetes associated with osteopenia. After 1 yr of therapy with cyclical etidronate (200 mg/day for 2 weeks every 3 months), intima-media thickness showed a decrease (mean ± SE, -0.038 ± 0.011 mm), which was significantly different from a change in 57 control subjects (0.023 ± 0.015 mm; *P *< 0.005). Cardiovascular parameters were not changed after etidronate treatment. The authors concluded that etidronate in clinical dosage may have an antiatherogenic action, at least in type 2 diabetes [123]. In another study, administration of ethane-1-hydroxy-1,1-diphosphonate (EHDP) to swine with pre-established atherosclerosis resulted in lower lesion calcium concentration, smaller lesions and a decrease in the area of lesions involved in necrosis [124].

## Atherosclerosis, plant polyphenols and fatty acids

Cupric-ion-oxidized LDL (CuLDL) or endothelial cell-oxidized LDL (ELDL) induces the activation by Tyr-phosphorylation of JAK2, one of the Janus kinase involved upstream of STATs in the JAK/STAT pathway of cytokine transduction. Oxidized LDL (OxLDL) also initiates STAT1 and STAT3 Tyr-phosphorylation and translocation to the nucleus, with a more marked effect for the extensively modified CuLDL. In one study, Genistein, a nonspecific Tyr-kinase inhibitor, and AG490, a specific inhibitor of JAKs, markedly prevented the CuLDL-induced enhancement of STAT1 and STAT3 Tyr-phosphorylation and DNA-binding activity, suggesting that JAKs are the main kinases involved in STATs' activation by oxidized LDL [125]. The effect of genistein on aortic atherosclerosis was studied in New Zealand White rabbits. After provocation of atherosclerosis with hyperlipidemic diet, the rabbits were divided as hyperlipidemic diet group (HD), normal diet group (ND) and hyperlipidemic plus genistein diet group (HD + genistein) for 4 and half months. The average cross sectional area of atherosclerotic lesion was 0.269 mm2 after provocation. The lesion was progressed by continuous hyperlipidemic diet (10.06 mm2) but was increased mildly by genistein (0.997 mm2), and decreased by normal diet [126]. Angiotensin II (Ang II) plays an important role in atherogenesis. One study investigated the effect of Ang II on the production of interleukin-6 (IL-6) in rat vascular smooth muscle cells. Ang II significantly increased the expression of IL-6 mRNA and protein in a dose-dependent manner (10(-10) to 10(-6) mol/L). The expression of IL-6 mRNA induced by Ang II was completely blocked by an Ang II type 1 receptor antagonist, CV11974. Inhibition of tyrosine kinase with genistein, and inhibition of mitogen-activated protein kinase with PD98059 completely abolished the effect of Ang II [127]. The potent endothelium-derived vasoactive factor endothelin-1 (ET-1) has been implicated in the pathophysiology of atherosclerosis and its complications. ET-1 stimulates the formation of proinflammatory cytokines including Interleukin-6 and tumor necrosis factor alpha (TNF alpha) [128]. In one study ET-1 transiently increased IL-6 mRNA compatible with regulation of IL-6 release at the pretranslational level. Electrophoretic mobility shift assays demonstrated time- and concentration-dependent activation of the proinflammatory transcription factor nuclear factor-kappaB (NF-kappaB) in ET-1-stimulated human vascular SMC. A decoy oligodeoxynucleotide bearing the NF-kappaB binding site inhibited ET-1-stimulated IL-6 release to a great extent suggesting that this transcription factor plays a key role for cytokine production elicited by ET-1 [129].

## Type 2 diabetes and Interleukin 6

Circulating levels of interleukin-6 (IL-6) are raised in insulin resistant states such as obesity, impaired glucose tolerance (IGT), and type 2 diabetes mellitus (DM). Growing evidence suggests that IL-6 is not only produced by fat cells but is also capable of inducing insulin resistance in these cells. The expected result of this in vivo, would be to increase adipose mass and subsequently body mass index (BMI). The IL-6 -174G > C common functional gene variant has consistently been associated with increased plasma IL-6, insulin resistance, and increased cardiovascular risk [130]. In The Women's Health Study (an ongoing US primary prevention, randomized clinical trial initiated in 1992), the authors determined whether elevated levels of the inflammatory markers interleukin 6 (IL-6) and C-reactive protein (CRP) are associated with development of type 2 DM in healthy middle-aged women. From a nationwide cohort of 27 628 women free of diagnosed DM, cardiovascular disease, and cancer at baseline, 188 women who developed diagnosed DM over a 4-year follow-up period were defined as cases and matched by age and fasting status with 362 disease-free controls. Study results showed that baseline levels of IL-6 (P < .001) and CRP (P < .001) were significantly higher among cases than among controls. The relative risks of future DM for women in the highest vs lowest quartile of these inflammatory markers were 7.5 for IL-6 (95% confidence interval [CI], 3.7–15.4) and 15.7 for CRP (95% CI, 6.5–37.9). Positive associations persisted after adjustment for body mass index, family history of diabetes, smoking, exercise, use of alcohol, and hormone replacement therapy. The authors concluded that elevated levels of CRP and IL-6 predict the development of type 2 DM, and the data support a possible role for inflammation in diabetogenesis.

## Type 2 diabetes and bisphosphonates

Advanced glycation end products (AGE), senescent macroprotein derivatives form at an accelerated rate in diabetes and induce angiogenesis through overgeneration of autocrine vascular endothelial growth factor (VEGF). In one study, incadronate disodium, a nitrogen-containing bisphosphonate, was found to completely inhibit AGE-induced increase in DNA synthesis as well as tube formation of human microvascular endothelial cells (EC). Furthermore, incadronate disodium significantly prevented transcriptional activation of nuclear factor-kappaB and activator protein-1 and the subsequent up-regulation of VEGF mRNA levels in AGE-exposed EC. Farnesyl pyrophosphate, but not geranylgeranyl pyrophosphate, was found to completely reverse the anti-angiogenic effects of incadronate disodium on EC. These results suggest that incadronate disodium could block the AGE-signaling pathway in microvascular EC through inhibition of protein farnesylation [131,132]. In another study, the bisphosphonate, pamidronate, given as a single dose led to a reduction in bone turnover, symptoms and disease activity in diabetic patients with active Charcot neuroarthropathy [133].

## Type 2 diabetes and statins

In West of Scotland Coronary Prevention Study (WOSCOPS) [134], development of type 2 diabetes mellitus (DM) was found to decrease by 30% in pravastatin-treated patients. One study investigated the effects of an HMG-CoA reductase inhibitor, atorvastatin, on insulin sensitization in performed in chow fed Zucker lean and fatty rats treated with atorvastatin 50 mg/kg/day (ATORVA_50) and results were compared to Zucker lean and fatty rats treated with drug vehicle only (CONT). Treatment with atorvastatin resulted in a dose-dependent improvement in whole body insulin sensitivity in both lean and fatty rats, with an approximately two-fold increase in glucose infusion rate and glucose disposal (Rd) in ATORVA_50 versus CONT (p < 0.01) [135]. Another study investigated the effects of atorvastatin on the glucose metabolism and insulin resistance of KK/Ay mice, an animal model of type 2 diabetes, were investigated. Atorvastatin significantly decreased the non-HDL-cholesterol level in the oral glucose tolerance test, inhibited increase in the 30-min glucose level, decreased plasma insulin levels before and 30 and 60 minutes after glucose loading, and decreased the insulin resistance index, compared with corresponding values in controls, indicating that atorvastatin appeared to improve glucose metabolism by improving insulin resistance [136].

## Type 2 diabetes, plant polyphenols and fatty acids

Nutritional intervention studies performed in animals and humans suggest that the ingestion of soy protein associated with isoflavones and flaxseed rich inlignans improves glucose control and insulin resistance. In animal models of obesity and diabetes, soy protein has been shown to reduce serum insulin and insulin resistance. In studies of human subjects with or without diabetes, soy protein also appears to moderate hyperglycemia and reduce body weight, hyperlipidemia, and hyperinsulinemia, supporting its beneficial effects on obesity and diabetes [137]. Recent studies have provided evidence that soy consumption alleviates some of the symptoms associated with Type 2 diabetes such as insulin resistance and glycemic control [138,139]. Isoflavones may improve lipid and glucose metabolism by acting as an antidiabetic PPAR agonist [140] The beta subunit of the signalsome – IKKbeta, a crucial catalyst of NF-kappaB activation – is an obligate mediator of the disruption of insulin signaling induced by excessive exposure of tissues to free fatty acids and by hypertrophy of adipocytes. IKKbeta plays a crucial role, not only in the induction of insulin resistance, but also atherogenesis, a host of inflammatory disorders, and the survival and spread of cancer. The polyphenols resveratrol and silibinin. inhibit or suppress the activation of IKKbeta [141]. Epidemiologic studies have reported a lower prevalence of impaired glucose tolerance and type 2 diabetes in populations consuming large amounts of the n-3 long-chain polyunsaturated fatty acids (n-3 LC-PUFAs) found mainly in fish [142].

## Osteoporosis and Interleukin 6

Osteoporosis is a condition that is common with aging and especially in post-menopausal women. The etiology has often been ascribed to abnormalities in calcium metabolism. However many patients with osteopenia/osteoporosis have in common pain and inflammation and many inflammatory pain syndromes have osteopenia/osteoporosis as an accompanying feature [143]. Inflammatory joint disease, particularly rheumatoid arthritis [144], is associated with bone resorption and increased synovial fluid levels of IL-6 [145]. Another example is the osteoporosis that is often present in Complex Regional Pain Syndrome/Reflex sympathetic dystrophy (CRPS-I/RSD) [146]. Interleukin-6 mediated inflammation has been shown to contribute to the process of bone remodeling. This it does by stimulating osteoclastogenesis and osteoclast activity [147]. Elevated levels of Interleukin-6 have been observed in conditions of rapid skeletal turnover and hypercalcemia as in Paget's disease and multiple myeloma [148]. In multiple myeloma, radiologic examinations reveals osteolytic lesion and the most common finding is diffuse osteopenia [149]. Adhesion of multiple myeloma cells to stromal cells triggers IL-6 secretion by the stromal cells [150]. This results in increased osteoclastic activity that in turn results in osteoporosis, painful osteolytic lesions and hypercalcemia characteristic of multiple myeloma [151]. In their youth, women are protected from osteoporosis because of the presence of sufficient levels of estrogen. Estrogen blocks the osteoblast's synthesis of Interleukin 6. Estrogen may also antagonize the interleukin 6 receptors. Decline in estrogen production is often associated with osteopenia/osteoporosis in postmenopausal women. Estrogen's ability to repress IL-6 expression was first recognized in human endometrial stromal cells [152]. Additional clues came from the observations that menopause or ovariectomy resulted in increased IL-6 serum levels [153], increased IL-6 mRNA levels in bone cells [154], and increased IL-6 secretion by mononuclear cells [155-157]. Further evidence for estrogen's ability to repress IL-6 expression is derived from studies, which demonstrated that estradiol inhibits bone marrow stromal cell and osteoblastic cell IL-6 protein and mRNA production *in vitro *[158] and that estradiol was as effective as neutralizing antibody to IL-6 in suppressing osteoclast development in murine bone cell cultures [159]or in ovariectomized mice [160].

## Osteoporosis and bisphosphonates

Bisphosphonates are inorganic chemical compounds that bind to hydroxyapatite in bone and prevent osteoclastic absorption of bone. Nitrogen-containing bisphosphonates (N-BPs) are potent inhibitors of bone resorption widely used in the treatment of osteoporosis and other bone degrading disorders including Paget's disease of bone, hypercalcemia associated with malignancy, metastatic bone diseases, such as breast cancer, multiple myeloma, and arthritis [161,162]. At the tissue level, N-BPs reduce bone turnover and increase bone mass and mineralization. This is measured clinically as an increase in bone mineral density and bone strength and a decrease in fracture risk. N-BPs localize preferentially at sites of bone resorption, where mineral is exposed, are taken up by ostoclasts and inhibit osteoclastic activity. At the molecular level, N-BPs inhibit an enzyme in the cholesterol synthesis pathway, farnesyl diphosphate synthase. As a result, there is a reduction in the lipid geranylgeranyl diphosphate, which prenylates GTPases required for cytoskeletal organization and vesicular traffic in the osteoclast, leading to osteoclast inactivation [163,164].

## Osteoporosis and statins

3-hydroxy-3-methylglutaryl coenzyme A reductase inhibitors (statins) have been shown to stimulate bone formation in laboratory studies, both in vitro and in vivo. Statin use in most, but not all observational studies is associated with a reduced risk of fracture, particularly hip fracture, even after adjustment for the confounding effects of age, weight and other medication use. This beneficial effect has not been observed in clinical trials designed to assess cardiovascular endpoints [165]. Men using statin drugs are more likely to have a greater BMD of the spine (p < 0.005), and men who receive statin drugs for more than 2 yr are approximately half as likely to develop osteoporosis. A similar effect is observed in women taking statins for any length of time [166]. Statin use in women is associated with a 3% greater adjusted BMD at the femoral neck, and BMD tends to be greater at the spine and whole body [167]. Nitrogen-containing bisphosphonate drugs inhibit the mevalonate pathway, preventing the production of isoprenoids, which consequently results in the inhibition of osteoclast formation and osteoclast function. Statins decrease the hepatic biosynthesis of cholesterol by blocking the mevalonate pathway, and can affect bone metabolism in vivo through effects on osteoclastic bone resorption. The ability of statin compounds to inhibit bone resorption is directly related to HMG-CoA reductase activity [168].

## Osteoporosis, plant polyphenols, and fatty acids

Dietary supplementation with soybean isoflavone can prevent postmenopausal bone loss. In one study, postmenopausal women (n = 19), mean age 70.6 +/- 6.3 years and mean time since menopause 19.1 +/- 5.5 years, were given isoflavone supplements for 6 months. There was a 37% decrease in urinary concentrations of type 1 collagen alpha1-chain helical peptide (HP), a marker of bone resorption, during the isoflavone supplementation compared with baseline (p < 0.05) and a significant difference in mean (SE) HP excretion levels when isoflavone was compared with placebo (43.4 +/- 5.2 vs. 56.3 +/- 7.2 microg/mmol creatinine [cr], p < 0.05). With isoflavone supplementation, mean spine BMD at L2 and L3 was significantly greater when treatment was compared with control, with a difference between means of 0.03 +/- 0.04 g and 0.03 +/- 0.04 g (p < 0.05), respectively. There were nonsignificant increases from baseline for total spine BMC (3.5%), total spine BMD (1%), total hip BMC (3.6%), and total hip BMD (1.3%) with the isoflavone treatment [169]. Data from a randomized, double-blind, placebo-controlled, year long clinical trial has also suggested that supplementation with the dietary phytoestrogen genistein (54 mg/day) may be as effective as hormone replacement therapy in attenuating menopause-related bone loss [170].

Beneficial effects of omega 3 fatty acids on bone mineral density have been reported in rats and humans. In one study, sham and ovariectomized (OVX) mice were fed diets containing either 5% corn oil (CO), rich in omega-6 fatty acids or 5% fish oil (FO), rich in omega-3 fatty acids. Bone mineral density was analyzed by DXA. The serum lipid profile was analyzed by gas chromatography. Receptor activator of NF-kappaB ligand (RANKL) expression and cytokine production in activated T-cells were analyzed by flow cytometry and ELISA, respectively. Significantly increased bone mineral density loss (20% in distal left femur and 22.6% in lumbar vertebrae) was observed in OVX mice fed CO, whereas FO-fed mice showed only 10% and no change, respectively. Bone mineral density loss was correlated with increased RANKL expression in activated CD4+ T-cells from CO-fed OVX mice, but there was no change in FO-fed mice [171].

## Aging, age-related disorders, and Interleukin 6

Evidence has linked IL-10 and IL-6 cytokine polymorphisms to longevity. Individuals who are genetically predisposed to produce high levels of IL-6 have a reduced capacity to reach the extreme limits of human life, whereas the high IL-10-producer genotype is increased among centenarians [172].

Telomere length is linked to age-associated diseases, with shorter telomeres in blood associated with an increased probability of mortality from infection or heart disease. In patients with multiple myeloma (MM), telomere length (TL) of MM cells is significantly shorter than that of the patients' own leukocytes. In one study, TL negatively correlated with age and with interleukin-6 (IL-6) and beta2-microglobulin levels [173]. Overproduction of IL-6, a pro-inflammatory cytokine, is associated with a spectrum of age-related conditions including cardiovascular disease, osteoporosis, arthritis, type 2 diabetes, certain cancers, periodontal disease, frailty, and functional decline. To describe the pattern of change in IL-6 over 6 years among older adults undergoing a chronic stressor, this longitudinal community study assessed the relationship between chronic stress and IL-6 production in 119 men and women who were caregiving for a spouse with dementia and 106 noncaregivers, with a mean age at study entry of 70.58 (SD = 8.03) for the full sample. On entry into this portion of the longitudinal study, 28 of the caregivers' spouses had already died, and an additional 50 of the 119 spouses died during the 6 years of this study. Levels of IL-6 and health behaviors associated with IL-6 were measured across 6 years. Caregivers' average rate of increase in IL-6 was about four times as large as that of noncaregivers. Moreover, the mean annual changes in IL-6 among former caregivers did not differ from that of current caregivers even several years after the death of the impaired spouse. There were no systematic group differences in chronic health problems, medications, or health-relevant behaviors that might have accounted for caregivers' steeper IL-6 slope. These data provide evidence of a key mechanism through which chronic stressors may accelerate risk of a host of age-related diseases by prematurely aging the immune response [174]. Interleukin-6 is also a causative factor in other manifestations of aging. Wrinkles on the skin are a manifestation of aging. Excess sunlight, smoking, and exposure to wind, heat, and harsh chemicals causes the outer layers of the skin to thicken and cause skin to wrinkle, sag and become leathery. Ultraviolet (UV) radiation from the sun is widely considered as a major cause of human skin photoaging and skin cancer. IL-6 is produced by keratinocytes in vivo and in vitro and the release is enhanced by UV light. A study was performed to investigate the effect of a single UV dose eliciting moderate to severe sunburn reaction on the production of IL-6 in vivo. Plasma of UV-treated human subjects was evaluated for IL-6 activity by testing its capacity to induce the proliferation of an IL-6-dependent hybridoma cell line (B9). In contrast to plasma samples obtained before UV exposure, post-UV-specimens contained significant levels of IL-6 peaking at 12 h after UV irradiation. Plasma IL-6 activity was neutralized by an antiserum directed against recombinant human IL-6 [175]. UV radiation-induced proinflammatory cytokines mediated by NF-kappaB reportedly play important roles in sunburn, skin damage, premature aging, and increases the risk of developing melanomas and other types of skin cancer. In one study, immunohistochemical and Western blot analysis and ELISA indicated that both nuclear p65 and secreted IL-6 were significantly (p < 0.05) induced by UVB (20, 30 mJ/cm2) and UVA irradiation (10, 20 J/cm2). NF-kappaB nuclear translocation and IL-6 secretion induced by UVB and UVA were dramatically inhibited by treatment of EGCG [176]. Higher levels of the systemic inflammatory markers CRP and IL-6 are independently associated with progression of age-related macular degeneration (AMD) [177].

## Aging, age-related disorders, Interleukin-6 and gene therapy/modulation

Genetic polymorphisms involving a change of a single base, fromguanine to cytosine, at position – 174 in the 5' flankingregion of the interleukin-6 gene is of great importance becausethe G allele is associated with higher IL-6 production thanthe C allele. In vivo studies have found basal IL-6 levels to be twice ashigh in volunteers with the GG allele than in those with theCC allele. The polymorphism in the 5' flanking region, (an area important in theregulation of gene expression) alters the transcriptional response to stimuli such as LPS and IL-1 [178]. An increased frequency of an Xba I Restriction Fragment Length Polymorphism (RFLP, likelyto be due to 3' flanking region insertions, has been describedin some patients with SLE and elevated IL-6 levels [179]. Byusing polymerase chain reaction (PCR)-RFLP and sensitive polyacrylamide gel electrophoresis, an association between genotype for the 3' flanking region polymorphismand peak bone mineral density in women has been demonstrated [180]. Manipulating the genetic mechanismscontrolling the IL-6 levels and increasing the frequency of GG alleles in the population would prevent aging and age related diseases and be the key to eternal youth and immortality. Gene therapy will aim to provide for targeted gene transfer, controlled expression of the gene transferred and enhanced activity of the transferred gene product. An alternate means of gene therapy is gene modulation. In gene modulation, expression of an already expressed gene is increased by introducing exogenous normal genetic sequences and decreased by introducing antisense genes or gene fragments, or by introducing vectors that can produce ribozymes that can cleave specific mRNAs. Gene modulation can also be achieved by the introduction of exogenous normal genetic sequences that code for proteins that modulate the extent of gene expression, or affect the processing, assembly or secretion of gene products.

## Conclusion

In conclusion, we have described the biochemical pathway from cholesterol synthesis to interleukin 6 mediated inflammation. Interleukin 6 mediated inflammation is the gatekeeper and common causative factor for aging and age-related disorders including Atherosclerosis, Peripheral Vascular Disease, Coronary Artery Disease, Osteoporosis, Type 2 Diabetes, Dementia and Alzheimer's disease and some forms of Arthritis and Cancer. We have clarified the relationship between some of these common illnesses and we determine that pleiotropic effects of bisphosphonates, statins and polyphenolic compounds are mediated by inhibition of Interleukin 6 mediated inflammation.

Isoprenoids, which are intermediates, generated in the cholesterol biosynthesis pathway, may play a role as significant as the end product cholesterol, in activation of Interleukin 6 mediated inflammation. Isoprenoids are generated by endogenous cellular cholesterol synthesis in the body as well as by cholesterol synthesis in activated monocytes during the inflammatory response. However, isoprenoids are but one component of the signaling pathway for Interleukin 6 mediated inflammation.

Inhibition of the signal transduction pathway for Interleukin 6 mediated inflammation is key to the prevention and treatment of aging and age-related disorders including atherosclerosis, peripheral vascular disease, coronary artery disease, osteoporosis, type 2 diabetes, dementia, Alzheimer's disease and some forms of arthritis and cancer. Inhibition of Interleukin 6 mediated inflammation may be achieved indirectly through regulation of endogenous cholesterol synthesis and isoprenoid depletion or by direct inhibition of the interleukin-6 signal transduction pathway.

Statins, Bisphosphonates and Polyphenolic Compounds have similar mechanisms of action and act on similar diseases in the following ways:

1. Statins and Bisphosphonates inhibit the Mevalonate to Cholesterol conversion pathway and cause isoprenoid depletion; with inhibition of interleukin-6 inflammation. Statins inhibit the enzyme HMG-CoA reductase and Bisphosphonates inhibit the enzyme FPP Synthase. Polyphenolic Compounds inhibit multiple pathways of signal transduction for Interleukin 6 mediated inflammation including inhibition of tyrosine kinase activity, inhibition of activation of NF-κB and inhibition of activation of IKK complex.

2. Statins, Bisphosphonates and Polyphenolic Compounds inhibit the JAK/STAT3 signaling pathway for Interleukin 6 mediated inflammation.

3. Statins, Bisphosphonates and Polyphenolic Compounds have common pleiotropic effects and decrease the progression of atherosclerotic vascular disease and inhibit bone resorption.

4. Combination treatment with agents that inhibit different aspects of the signal transduction pathways for interleukin 6 mediated inflammation, including Statins, Bisphosphonates and Polyphenolic Compounds, will be transformational and have better efficacy with fewer side effects in the prevention and treatment of aging and age-related disorders including atherosclerosis, peripheral vascular disease, coronary artery disease, osteoporosis, type 2 diabetes, dementia and some forms of arthritis and tumors. Evidence of safety and efficacy of combination treatment with inhibitors of Interleukin-6 mediated inflammation should be sought from new clinical trials.

Statins, Bisphosphonates are just indirect inhibitors of Interleukin-6 inflammation but yet both class of drugs have enabled a significant decrease in mortality and morbidity from these common illnesses.

Epidemiological evidence suggests that increased consumption of plant derived polyphenolic compounds is associated with decrease in mortality and morbidity from these common illnesses. Newer therapies will include delivering by gene therapy or gene modulation variations and/or modifications of the interleukin-6 gene associated with decreased or absent IL-6 production. Newer drugs will include interleukin-6 inhibitor/antibody, interleukin-6 receptor inhibitor/antibody, interleukin-6 antisense oligonucleotide (ASON), gp130 protein inhibitor/antibody, tyrosine kinases inhibitors/antibodies, serine/threonine kinases inhibitors/antibodies, mitogen-activated protein (MAP) kinase inhibitors/antibodies, phosphatidylinositol 3-kinase (PI3K) inhibitors/antibodies, Nuclear factor κB (NF-κB) inhibitors/antibodies, IκB kinase (IKK) inhibitors/antibodies, activator protein-1 (AP-1) inhibitors/antibodies, STAT transcription factors inhibitors/antibodies, altered IL-6, partial peptides of IL-6 or IL-6 receptor, or SOCS (suppressors of cytokine signaling) protein, PPAR gamma and/or PPAR beta/delta activators/ligands or a functional fragment thereof.

The public health significance of such new drugs and gene therapy will be transformational.
